# Application of Inflatable Video-Assisted Mediastinoscopic Transhiatal Esophagectomy in Individualized Treatment of Esophageal Cancer

**DOI:** 10.3390/biomedicines11102750

**Published:** 2023-10-11

**Authors:** Shangqi Song, Cheng Shen, Yang Hu, Yazhou He, Yong Yuan, Yuyang Xu

**Affiliations:** 1 Department of Thoracic Surgery, West China Hospital, Sichuan University, Chengdu 610041, China; sq_song@wchscu.cn (S.S.);; 2 Usher Institute of Population Health Sciences, The University of Edinburgh, Edinburgh EH8 9YL, UK

**Keywords:** esophageal neoplasms, inflatable mediastinoscopy, esophagectomy, complications, individualized management

## Abstract

Surgery is a crucial treatment option for patients with resectable esophageal cancer. The emergence of minimally invasive esophageal techniques has led to the popularity of video-assisted thoracoscopic esophagectomy, which has proven to be more advantageous than traditional thoracotomy. However, some patients with esophageal cancer may not benefit from this procedure. Individualized treatment plans may be necessary for patients with varying conditions and tolerances to anesthesia, making conventional surgical methods unsuitable. Inflatable video-assisted mediastinoscopic transhiatal esophagectomy (IVMTE) has emerged as a promising treatment option for esophageal cancer because it does not require one-lung ventilation, reduces postoperative complications, and expands surgical indications. This technique also provides surgical opportunities for patients with impaired pulmonary function or thoracic lesions. It is crucial to have a comprehensive understanding of the advancements and limitations of IVMTE to tailor treatment plans and improve outcomes in patients with esophageal cancer. Understanding the advantages and limitations of this surgical method will help specific patients with esophageal cancer. We conducted a thorough review of the relevant literature to examine the importance of IVMTE for individualized treatment of this disease.

## 1. Introduction

Esophageal cancer is the seventh most common malignant tumor worldwide and the sixth leading cause of cancer-related deaths among patients with cancer [1,2]. It has a relatively high incidence in China, with nearly 90% of its pathological types being squamous cell carcinoma [3,4]. However, esophageal cancer has a relatively poor prognosis, with a 5-year survival rate of <40% [5]. Surgery is still considered the primary option for potentially curative treatment [6,7].

In recent years, with the advancement of minimally invasive techniques, laparoscopic esophagectomy has been widely used, which can shorten the postoperative rehabilitation time and reduce the probability of complications compared to traditional thoracotomy [8]. In comparison to transthoracic approaches, such as video-assisted thoracoscopic esophagectomy (VATE), which relies heavily on one-lung ventilation, the video-assisted mediastinoscopic transhiatal esophagectomy (IVMTE) procedure reaches the middle and upper esophagus via the cervical approach, eliminating the need for chest wall incisions and one-lung ventilation. This surgical method significantly reduces postoperative pain and the impact on cardiovascular function, ultimately accelerating patients’ recovery [9]. Consequently, this minimally invasive transmediastinal approach has become a viable alternative to the traditional transthoracic esophagectomy [10,11]. In 1947, Lewis et al. firstly proposed transhiatal esophagectomy [12]. In 1990, Buess first reported on esophagectomy via mediastinoscopy, which improved the safety of the surgical procedure by providing direct vision [13]. Nevertheless, achieving systematic lymph node dissection remains challenging because of limited operating space. Subsequently, mediastinoscopy has been performed for esophageal cancer resection, and carbon dioxide is used to enlarge the space by creating a pneumomediastinum [14]. Fujiwara et al. proposed IVMTE in 2015, making this surgical approach mature and progressively promoted after gradual evolution and improvement [15]. However, the tunneled surgical approach also brings difficulties to operation, and whether an anatomical structure can be clearly exposed and lymph nodes can be thoroughly dissected under mediastinoscopy has once been questioned [16,17]. We consulted the relevant literature to elaborate on the clinical progress and existing limitations of IVMTE, especially in individualized treatment of esophageal cancer.

## 2. Indications for IVMTE of Esophageal Cancer

IVMTE is less invasive than video-assisted thoracoscopic esophagectomy (VATE) because it avoids one-lung ventilation and chest trauma. This is a viable option for patients who cannot tolerate thoracotomy or thoracoscopic surgery. Indications for IVMTE include advanced age, severe pleural adhesions from a prior chest surgery, pleurisy, and past pulmonary tuberculosis. It is also recommended for patients with emphysema who have an FEV1 < 70% and vital capacity < 80% [18]. Additionally, it is suitable for patients with confirmed esophageal cancer who can be treated with R0 resection after preoperative evaluation. Some researchers suggest that IVMTE can be used for early esophageal cancer (T1–2 stage, tumor diameter < 2 cm, well-differentiated, and no lymph node metastasis), whereas others believe that it can be expanded to mid-stage esophageal cancer (no more than T3N1M0 stage) [19]. A study conducted by Daiko et al. found that surgical indications for patients with impaired lung function and a high Charlson Comorbidity Index (CCI ≥ 3) were viable as long as they could tolerate two-lung ventilation [20]. Additionally, for older patients, surgical indications for esophageal cancer should be evaluated based on the patient’s physical condition, life expectancy, tumor stage, and personal preferences [21]. When assessing the risk of postoperative complications, scoring systems, such as the estimation of physiological ability and surgical stress (E-PASS), the controlling nutritional status (CONUT), and the risk calculators provided by the Japanese National Clinical Database, should be used appropriately [22]. For patients with esophageal cancer and impaired organ function, non-thoracic esophagectomy, such as mediastinoscopy, is a better option for minimizing surgical trauma and replacing traditional transthoracic esophagectomy [23,24]. Additionally, it is crucial to preserve the bronchial artery, thoracic duct, and azygos vein arch.

## 3. Contraindications for IVMTE of Esophageal Cancer

Contraindications for IVMTE include (1) no definite pathological diagnosis made preoperatively; (2) severe organ dysfunction; (3) presence of distant metastasis; (4) no organ replacement in the digestive tract; and (5) when performing IVMTE, it is important to avoid factors that may hinder exposure and mobilization due to limited operating space. These factors include severe spinal deformity, stage T4 tumors, large primary tumors, significant lymphadenopathy, distant lymph node metastasis, and tissue swelling and adhesions resulting from preoperative adjuvant chemotherapy or radiotherapy. This has been mentioned in previous relevant literature [25,26]. Some investigators have suggested that IVMTE may be a viable option if CT examination determines that the tumor is resectable, regardless of whether prior treatment has been administered [27,28]. The indications and contraindications for IVMTE of esophageal cancer are summarized in Table 1.

## 4. Surgical Methods

During the early stages of mediastinoscopy in esophagectomy, limitations in the device and technology prevented a clear exposure of the mediastinal anatomy. Therefore, only esophageal mobilization and lymph node sampling were performed, but complete lymph node dissection was not possible [29]. If intraoperative rapid frozen-section pathological examination suggests lymph node metastasis, it may be necessary to change the body position and perform transthoracic lymph node dissection. This limits the application and promotion of the surgical approach.

### 4.1. Conventional IVMTE Mode

In 2015, Fujiwara et al. [15] proposed that the combination of pneumatic mediastinoscopy and laparoscopic esophagectomy represents a mature surgical approach that is increasingly being adopted by multiple medical centers. The surgical procedure involves the steps described below.

#### 4.1.1. Cervical Procedures

The patient is positioned supine and administered general anesthesia with two-lung ventilation using a single-lumen endotracheal tube. A 4 cm incision is made on the left side of the neck, allowing access to the anterior cervical muscles. The sternocleidomastoid muscles are exposed along the medial side of the anterior cervical muscles. The cervical esophagus is mobilized, and the lymph nodes near the left recurrent laryngeal nerve (RLN) are dissected. A lap protector with an access port is inserted to seal the gap in the neck wound. This procedure results in the formation of a pneumomediastinum through inflation with carbon dioxide (8 mmHg, 1 mmHg = 0.133 kPa).

#### 4.1.2. Transcervical Mediastinal Procedures

These procedures are performed using a LigaSure Maryland jaw sealer, and a special retractor is used to retract the arteries and esophagus. The azygos vein can be preserved because it does not affect the exposure of the esophagus; however, it is often cut off using the transthoracic approach (especially the right transthoracic route). The lymph nodes are mobilized and dissected en bloc. Finally, a right neck incision is made to dissect the lymph nodes adjacent to the right RLN under direct vision.

#### 4.1.3. Abdominal Procedures

Carbon dioxide inflation (10 mmHg) is used for both transabdominal and transdiaphragmatic hiatus procedures. During abdominal surgery, the surgeon uses their left hand to assist the laparoscope and controls the stomach through a midline incision. In transesophageal hiatal surgery, the surgeon controls the esophagus and liver for hiatal dilation. The esophageal hiatus is opened by cutting the gastrosplenic ligament to allow entry into the mediastinum through the left diaphragmatic crus.

#### 4.1.4. Transabdominal Mediastinal Procedures

The esophagus is mobilized axially, and the subcarinal and bilateral main parabronchial lymph nodes are dissected en bloc. The stomach is completely dissociated, the left gastric blood vessels are severed, and the abdominal lymph nodes are dissected. Finally, the esophagus is transected through a left cervical incision. A tubular stomach is created and lifted to the neck for the esophageal anastomosis.

### 4.2. Modified IVMTE Mode

In contrast to Fujiwara et al. [15], who ultimately performed a right cervical incision, which was only used for right recurrent laryngeal paraneurysm lymph node dissection under direct vision, Daiko et al. [14,20] proposed bilateral mediastinoscopy-assisted transdiaphragmatic hiatal laparoscopic esophagectomy (BTCMATLE) by adding a right cervical incision. The procedure is described below.

#### 4.2.1. Cervical Procedures

A 4–5 cm incision is made 1 cm lateral to the bilateral sternocleidomastoid muscles to access the tissue space and dissect the cervical paraesophagus (No. 101 group) and most upper mediastinal lymph nodes, including the bilateral RLN lymph nodes (No. 106recR group and No. 106recL group). A lap protector is inserted into the cervical wound on both sides, the gap is closed with ports, two introducer sheaths are placed separately, a 5 mm flexible mediastinoscope lens and grasping forceps are placed on the left side, and a LigaSure Maryland jaw sealer and a retractor are placed on the right side.

#### 4.2.2. Mediastinal Procedures

The esophagus is first freed from the anterior portion and then advanced from right to left and downwards. Typically, it can be sufficiently mobilized to expose the left atrium, after which the posterior esophagus is mobilized along the descending aorta. Finally, the left side of the esophagus is mobilized to meet the abdominal approach at the level of the left atrium. Upper (No. 105 group), middle (No. 108 group), and anterior (No. 112aoa group) thoracic paraesophageal lymph node dissections are performed. For patients with mediastinal stenosis, the BTCMATLE had good operability and a stable surgical field of view.

### 4.3. West China Hospital IVMTE Model

**Figure 1:**
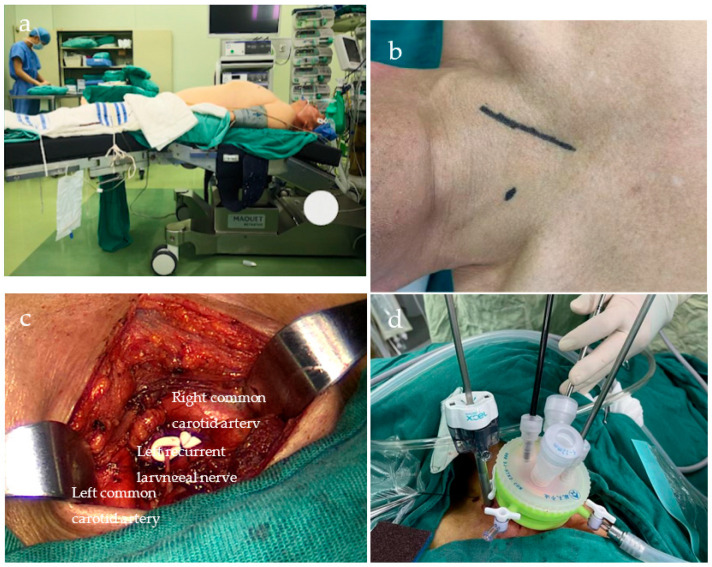
(**a**) Position of the patient, (**b**) cervical incision, (**c**) left cervical mobilization, and (**d**) dispensation of the cervical device.

**Figure 2:**
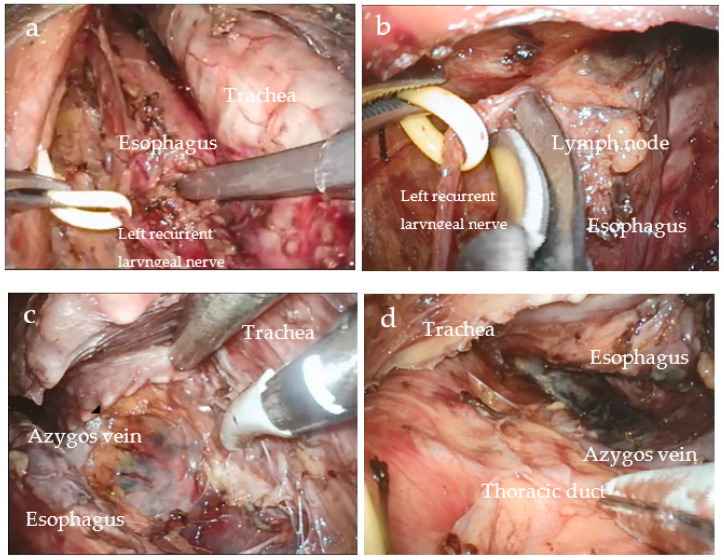
Transcervical view during esophageal mobilization. (**a**) Upper esophageal mobilization, (**b**) dissection of the lymph nodes around left RLN, (**c**) anterior mobilization of esophagus, and (**d**) posterior mobilization of esophagus.

Our center has also modified the surgical approach, with the left neck incision as the main operation path and the right minor incision as the auxiliary path. The surgical procedure is as follows: The patient is placed in a supine position (Figure 1a), and a 6–7 cm incision is made along the anterior border of the left sternocleidomastoid muscle (Figure 1b). The skin and subcutaneous tissues are carefully incised layer by layer. Muscle groups are gently separated. This allows visualization of the lower poles of the thyroid gland, trachea, and bilateral RLNs (Figure 1c). Additionally, bilateral cervical lymph nodes are dissected during the procedure. A small incision is made at the midpoint of the left lateral incision located at the anterior border of the right sternocleidomastoid muscle. A 5 mm trocar is then inserted, and a lap protector is placed in the left incision to establish a closed cavity. Finally, the mediastinum is inflated (pressure of 8 mmHg and flow of 10 L/min). An aspirator, mediastinoscope lens, and a LigaSure Maryland jaw sealer are placed on the left side, and a retractor is placed on the right side (Figure 1d). The esophageal wall is dissected in accordance with the sequence of “left—anterior—right—posterior” (Figure 2a). This is performed up to the carina level and is sometimes extended down to the inferior pulmonary vein level. During this process, various lymph nodes are also dissected, including the bilateral RLN, paraesophageal, tracheobronchial (No. 106tbL group), and subcarinal lymph nodes (Figure 2b). The authors concluded that dissecting the bilateral lymph nodes adjacent to the RLN is technically feasible through a left cervical incision under direct vision. By utilizing a suction apparatus and retractor, adequate exposure can be achieved while minimizing smoke interference, thereby decreasing surgical difficulty (Figure 2c,d). Morbidities associated with the IVMTE learning curve were analyzed. We found that improvements in the original technique allowed us to safely overcome the learning curve [30].

## 5. Perioperative Outcomes

Some references do not specify whether CO_2_ is used to expand the space during mediastinoscopic esophagectomy, but the statistical significance is still evident when compared to the transthoracic approach. Therefore, for this section, this article specifically refers to video-assisted mediastinoscopic thoracic esophagectomy (VMTE) instead of IVMTE.

### 5.1. Operation Time

Combining mediastinoscopy with laparoscopic surgery for esophageal cancer eliminates the need for changing body position and avoids cumbersome procedures, such as chest incisions and one-lung ventilation. Furthermore, this approach can be performed simultaneously by two separate medical teams, one for mediastinal surgery and the other for abdominal surgery, which significantly reduces operation time [31]. Sasaki et al. [32] compared data from 38 and 34 patients who underwent thoracoscopic and mediastinoscopic esophageal cancer surgeries, respectively. The results indicated that the mediastinoscopic group had significantly shorter operation time, mechanical ventilation time, and ICU stay.

### 5.2. Pulmonary Complications

Chen et al. [33] analyzed the data of 129 patients who underwent minimally invasive esophagectomy. Using the propensity score matching method, they matched 102 patients (51 pairs) and found that patients in the mediastinoscopy group had a lower incidence of postoperative pneumonia and atelectasis than those in the thoracoscopic group (*p* < 0.05). According to Tandon et al. [34], prolonged thoracoscopic one-lung ventilation and lung collapse can directly lead to acute respiratory distress syndrome and severe oxidative stress. As a result, the authors argue that VATE may not be appropriate for certain patients with cardiopulmonary dysfunction and that VMTE could be a viable alternative.

Poor respiratory function is a risk factor influencing the drainage volume of pleural effusion. Although VMTE does not involve the manipulation of the pleural cavity, postoperative pleural effusion is common, particularly on the left side [35]. Hamada et al. [35] analyzed data from 118 patients with VMTE and found that 34 patients required a single thoracic drainage and 41 patients required multiple postoperative drainages. They also found that the fold increase in CRP levels was significantly higher in patients with multiple DPEs than in patients with a single DPE on POD3 (*p* = 0.02). The same trend was confirmed on POD5 (*p* = 0.06). Hisakura et al. [36] attempted to incise the left mediastinal pleura during VMTE and indwell a 19Fr drainage tube in the left chest cavity through the esophageal hiatus and the abdominal wall. The rate and probability of postoperative thoracentesis drainage were significantly lower than in patients without drainage (*p* < 0.01). Hamada et al. suggested that changes in CRP levels be tested as an indicator of DPE.

### 5.3. RLN Injury

Verifying the presence of hoarseness following esophageal cancer surgery is crucial for determining whether the RLN is injured during the procedure. Jin et al. [27] analyzed data from 49 patients who underwent minimally invasive surgery for esophageal cancer. Of these, 30 and 19 underwent VATE and VMTE, respectively. The results revealed a significantly higher likelihood of postoperative hoarseness in the VMTE group than that in the VATE group. Similar results were reported by Sasaki et al. [32]. Because of the customary practice of making a left cervical incision, many patients experienced left RLN palsy. To address this issue, we perform intraoperative nerve monitoring to locate the RLN. This technique enables surgeons to make real-time adjustments during the procedure based on monitoring feedback to prevent nerve damage caused by compression, dragging, thermal injury, or ischemia [37]. However, it prolongs the operative time and increases patient medical costs. Excessive reliance on intraoperative nerve monitoring also affects the improvement of surgeons’ surgical skills. Kitagawa et al. [38] demonstrated that patients with esophageal carcinoma and a narrow mediastinum had a greater likelihood of experiencing postoperative hoarseness. Additionally, their study found that a sternal-to-vertebral distance of less than 45 mm was an independent risk factor for postoperative hoarseness. Daiko et al. [20] reduced the incidence of RLN palsy from 63% to 13% by altering the surgical sequence and dissecting the left RLN lymph nodes, thereby reducing direct collision of the surgical instrument arm with the RLN. Additionally, measures were taken to protect the nerves by utilizing the surrounding adipose tissue. In patients with early esophageal cancer, skeletalization of the RLN can easily cause damage [39]. Therefore, intraoperative management of the RLN should be minimized.

### 5.4. Number of Dissected Lymph Nodes

Existing guidelines advocate complete lymph node dissection to achieve more accurate lymph node staging and better long-term prognosis [40,41]. However, the mediastinal approach has received criticism for incomplete lymph node dissection and is only used for mediastinal lymph node biopsies and in early-stage (stage N0) patients who cannot tolerate one-lung ventilation. The available results indicate that mediastinoscopic lymph node dissection is inferior to thoracoscopic mediastinal lymph node dissection in terms of the number of stations [42,43].

Furthermore, some investigators have suggested that thoracoscopy is superior to RLN dissection [44]. Since Fujiwara reported that this surgical approach has continuously improved after en bloc lymph node dissection and radical resection of esophageal cancer under pneumomediastinum, an increasing number of thoracic surgeons have recognized the feasibility of extensive lymph node dissection under mediastinoscopy, which is comparable to thoracoscopy and reflects the advantages in dissecting the left RLN lymph nodes [15].

Using a right neck incision, in addition to a single left neck incision, not only increases the effectiveness of lymph node dissection near the right RLN but also circumvents the blockage caused by the aortic arch, minimizes the arrow effect, and allows for better visualization of the lymph nodes from the aortic arch to the left tracheobronchial nerve [41]. Simultaneously, it requires additional instruments for traction, and performing traction of the trachea ventrally facilitates lymph node exposure and thorough lymph node dissection [45,46]. It is also effective for left tracheobronchial lymph nodes that could not be removed under a single incision on the left side (No. 106 tbL group) [47,48].

Our author center has had the same experience after the implementation of IVMTE. However, we recommend dissection of right recurrent laryngeal lymph nodes under direct vision through a left cervical incision. This approach requires only a small incision in the right neck, but it provides the same surgical experience as a bilateral cervical incision. The results indicate that there is no significant difference in the number of dissected lymph nodes or the positive rate of lymph nodes between mediastinoscopy and thoracoscopy.

### 5.5. Hemorrhage

VMTE is less invasive and is associated with less intraoperative blood loss than VATE. Owing to the limited operating space, even a small amount of bleeding can significantly affect the surgical field during mediastinoscopy. Managing bleeding during this procedure can be particularly challenging. The most frequently encountered complication of esophagotracheal space dissection is bronchial artery injury [17]. Fortunately, minor bleeding can often be managed by using energy devices or gauze packing. However, in cases of more severe bleeding, a prompt switch to thoracoscopy or thoracotomy may be necessary to save the patient’s life, if other attempts to stop the bleeding are unsuccessful. Japanese scholars have proposed preoperative vascular three-dimensional reconstruction to assess the course pattern of the bilateral bronchial arteries [14]. During mediastinoscopy, the right bronchial artery, originating from the intercostal bronchial trunk artery, can be successfully identified in most patients. Surgical videos also show that other types of right bronchial arteries, such as those originating from the common trunk of the bronchial arteries or directly from the aorta, are relatively easy to identify [48]. Subcarinal lymph node dissection often causes bleeding when performed along the tracheal bifurcation. To avoid bleeding, the main bronchial lymph nodes are divided from the bilateral periphery into the carina and subcarinal lymph nodes [11].

The authors concluded that the most important aspect of the surgery is that the azygos vein must be dissected under direct vision and, if necessary, the azygos vein can be sutured, repaired, or directly ligated for hemostasis. Aortic injuries are mostly caused by overheating of energy devices or tumor invasion. The selection of energy devices with good temperature-control performance is particularly critical. Preoperative assessment of tumor lesions is also very important, and most investigators believe that T4 tumors are unsuitable for VMTE.

### 5.6. Thoracic Duct Injury

The probability of thoracic duct injury is relatively low, at approximately 1.67% [36]. The most common site of injury is the fifth thoracic vertebra, where the thoracic duct crosses the midline anterior to the vertebral body. Notably, the thoracic duct anatomy is highly variable, and its branches may occasionally be compromised. A preoperative high-fat diet can effectively fill the thoracic duct, allowing better visualization during surgery and reducing the likelihood of injury. Routine ligation of the thoracic duct is not recommended. When the thoracic duct is suspected to be injured, the main trunk and branches must be ligated. When the wound seeps or there are vascular-like structures injured, it often indicates thoracic duct injury. This situation is particularly noticeable when a high-fat diet, such as milk or olive oil, is consumed before surgery.

### 5.7. Anastomotic Leakage

Shi et al. [49] conducted a prospective, multicenter, open-label, randomized controlled trial including 200 patients with esophageal cancer, of whom 100 patients were in the thoracoscopic group and 100 patients were in the mediastinoscopic group. The results showed that there was no significant difference in the incidence of anastomotic leakage between the two groups (VATE vs. VMTE: 16% vs. 11%, *p* = 0.408).

### 5.8. Inflammatory Responses

Sasaki et al. [32] conducted a study that showed that the VMTE group had a significantly lower white blood cell count on POD5 compared to the VATE group (*p* = 0.0374). Additionally, the VMTE group had lower serum C-reactive protein (CRP) levels on POD3 than the VATE group (*p* = 0.0086). Continuous inflammatory stimulation can lead to increased pleural effusion. Hamada et al. [35] found that an increased white blood cell count is related to increased pleural effusion and suggested that changes in CRP levels can be used as an indicator of pleural effusion drainage.

### 5.9. Definition of Complications

The Clavien–Dindo classification, CTCAE, or the complication definitions proposed by the ECCG were utilized to classify the severity of complications in nine reports (Table 2).

## 6. Long-Term Outcomes

In the study conducted by Sasaki et al. [32], data from 72 patients who underwent minimally invasive esophagectomy were analyzed. Of these, 38 and 34 underwent thoracoscopy and mediastinoscopy, respectively. The results showed that the median survival time for the thoracoscopy and mediastinoscopy groups was 36.0 and 27.5 months, respectively, and there was no significant difference between the two groups. In addition, there was no significant difference in the overall survival rate between the two groups (*p* = 0.8896). Chen et al. [33] conducted a propensity score matching study of thoracoscopy versus mediastinoscopy and showed that there was no significant difference in recurrence-free survival between the thoracoscopy and mediastinoscopy groups (82.4% vs. 86.3%; *p* = 0.63).

## 7. Potential Advantages and Challenges of IVMTE

### 7.1. Advantages of IVMTE

Surgery is the mainstay treatment for esophageal cancer; however, the traditional transthoracic approach requires maintenance of one-lung ventilation and is difficult to perform in some patients with esophageal cancer who experience poor cardiopulmonary function and thoracic atresia. The mediastinal approach solves these problems and provides patients with surgical opportunities. However, the operative space under a mediastinoscope is narrow, and the anatomical structure is difficult to observe. Whether extensive lymph node dissection can be performed has always been debated. Pneumatic mediastinoscopy artificially creates mediastinal emphysema, expands the mediastinal space, and provides better space and vision for mediastinoscopy.

In summary, we believe that IVMTE has the following advantages:

- One-lung ventilation is not required, and its advantages are more evident in some patients with cardiopulmonary insufficiency.
- No damage to the intercostal nerves, good patient comfort, and fast postoperative recovery.
- There is no need to change the body position, and mediastinal and abdominal surgeries can be performed simultaneously, which shortens the operation and anesthesia time.
- The azygos vein and bronchial artery are preserved during surgery, thus preventing liver injury in patients with hepatic insufficiency and reducing the probability of postoperative coughing.
- It is suitable for patients with chest wall lesions who present difficulty for the surgical device when entering the chest through the intercostal space.

### 7.2. Challenges faced by IVMTE

According to the current literature, most surgical indications are limited to T3 (TNM-staging) and below, and when the tumor volume is too large to squeeze into the limited operating space, which causes difficulties during operation and affects patient safety. Whether VMTE is safe and effective for patient downstaging after neoadjuvant therapy due to tissue swelling and adhesions has not been reported in the literature.

Mediastinoscopy is a common surgical approach that involves a single incision on the left side of the neck. However, this method can be challenging when it comes to exposing certain structures. For instance, lymph nodes in the group 106 tbL (left tracheobronchial lymph nodes) are frequently obstructed by the aortic arch and left main bronchus, making dissection challenging. However, by utilizing bilateral neck incisions and a small incision on the right, along with additional traction instruments, satisfactory exposure can be achieved, resulting in more comprehensive lymph node dissection.

The narrow space in which surgery can occur can be hindered by smoking and wound bleeding. To overcome this, Maryland pliers are suitable energy devices to use because of their relatively low temperatures, low thermal diffusion, lack of carbonization, and less smoke. It is important to take care during the operation and pick up an appropriate amount of tissue to avoid continuous coagulation for an extended period. While maintaining effective mediastinal emphysema, it is important to avoid damaging the mediastinal pleura. To achieve this, the authors suggested using a laparoscopic suction device. This device has the following two purposes: First, it could be used as a pulling device to provide continuous stretching and exposure. Second, it can function as a suction device to continuously discharge smoke and remove oozing blood in cases of bleeding. This allows a clear view of the operation, enabling the surgeon to operate precisely.

VMTE may cause damage to the RLN due to instrument compression, drag, thermal injury, and ischemia after skeletalization. In addition to paying attention to gentle surgical operations and avoiding the use of energy devices, some researchers have improved surgical methods, such as intraoperative nerve monitoring, changing the order of surgery, cleaning the lymph nodes next to the left RLN, and marking the left RLN with a rubber ring to reduce the risk of RLN paralysis. Some researchers have suggested that for early-stage patients, the lymph nodes next to the RLN should be subjected to quick frozen-section pathological examination during surgery. If the result is negative, only lymph node sampling can be performed to reduce damage to the RLN [33].

The eighth edition of the AJCC Staging Manual suggests that, to ensure that patients will receive the maximum survival benefit, at least 10, 20, and 30 lymph nodes should be dissected for pathological T1, T2, and T3–4 tumors, respectively. The NCCN guidelines state that a minimum of 15 lymph nodes should be dissected in patients undergoing esophagectomy without preoperative chemoradiotherapy to achieve appropriate lymph node staging [42].

### 7.3. Disadvantages of IVMTE

- The narrow operating space poses a risk of damage to the surrounding tissues and limits the ability to handle unforeseen circumstances during surgery.
- There remains a gap between lymph node dissection and VATE.
- Compared to VATE, the risk of RLN injury is higher.
- The absence of a chest drainage tube increases the likelihood of postoperative pleural effusion, which may necessitate a thoracentesis.

## 8. Conclusions

Although VMTE has many advantages, several problems need to be solved. First, it is necessary to determine whether intraoperative lymph nodes require rapid frozen-section pathological examination. If the test results are positive, the question arises as to whether thoracoscopic surgery should be performed for more thorough lymph node dissection. Second, owing to its dependence on specialized equipment and technical expertise, VMTE is currently only available in selected medical centers. Simplifying the operational process is conducive to its popularization. Third, it is important to determine whether VMTE is a viable option for patients with esophageal cancer who have undergone neoadjuvant therapy for downstaging. Additionally, assessing whether VMTE can achieve safety and efficacy comparable to those of VATE is crucial. Fourth, establishing a viable clinical evaluation system and effectively screening potential beneficiaries are essential. Finally, it is imperative to determine whether there is a response plan for possible mediastinoscopic accidents during operation. We argue that the majority of the current research on VMTE consists of retrospective studies with limited sample sizes. To better assess this surgical method’s safety and effectiveness, there is a need for large-scale, multi-center clinical trials.

## Figures and Tables

**Table 1:** Indications and contraindications for IVMTE of esophageal cancer.

	IVMTE	VATE
Indications	Advanced age	Any age
	Severe pleural adhesion	Except severe pleural adhesion
	Emphysema with FEV1 < 70% and vital capacity < 80%	Sufficient lung function to tolerate one-lung ventilation
	Histopathology confirms esophageal cancer that can be treated with R0 resection	
Contraindications	No definite pathological diagnosis	
	Severe organ dysfunction	
	Presence of distant metastasis	
	Absence of replacement organs for the digestive tract	
	Factors that cause tight operating space: severe spinal deformity, tumor stage T4, large primary tumor, significant lymphadenopathy, distant lymph node metastasis, and tissue swelling and adhesion resulting from adjuvant chemotherapy or radiotherapy	Unresectable with invasion of adjacent tissues

**Table 2:** Postoperative complications of IVMTE.

						Pathology (Cases)				Complication (%)				
No.	Authors	Year	Ref	Country	N (Cases)	SCC	Adeno.	Other	Definition of Complication	Pneumonia	Arrhythmia	Leakage	Chylothorax	RLNP
1	Feng	2012	[50]	China	27	27	0	0		25.9		18.5	0	18.5
2	Wang	2015	[51]	China	194	194	0	0		6.2	3.6	4.6		4.6
3	Fujiwara	2017	[52]	Japan	60	58	2	0	CD, ECCG	6.7		15	0	33.3
4	Chen	2022	[33]	China	59	59	0	0		8.5		13.6	1.7	
5	Sasaki	2022	[32]	Japan	34	32	0	2	CD	17.7		17.7	5.9	38.2
6	Shi	2022	[49]	China	100	100	0	0	ECCG	7	6	11		12
7	Huang	2023	[8]	China	38				CD	7.9	7.9	5.3	2.6	
8	wang	2023	[53]	China	30	30	0	0	CD	10	6.7	6.7	0	3.3
9	Zhang	2023	[54]	China	106	106	0	0	CD	3.77		14.15	2.83	1.89

Abbreviations: SCC, squamous cell carcinoma; Adeno., adenocarcinoma; CD, Clavien–Dindo classification; CTCAE, Common Terminology Criteria for Adverse Events; ECCG, complication definitions by the Esophageal Complications Consensus Group; RLNP, recurrent laryngeal nerve palsy.

## Data Availability

Not applicable.
